# Supplementary material for: Time to Death and Nursing Home Admission in Older Adults with Hip Fracture: A Retrospective Cohort Study
Source: J Clin Med. 2025 Dec 4;14(23):8603. doi: 10.3390/jcm14238603 (PMC12693230; doi:10.3390/jcm14238603)
Supplement: Supplementary file 1 [file jcm-14-08603-s001.zip › jcm-3989248-supplementary.pdf]

Supplementary Table S1. STROBE Statement—Checklist of items that should be included in reports of ***cohort studies***

	Item No	Recommendation	Page No
**Title and abstract**	1	(*a*) Indicate the study's design with a commonly used term in the title or the abstract	1, abstract
		(*b*) Provide in the abstract an informative and balanced summary of what was done and what was found	abstract
**Introduction**			
Background/rationale	2	Explain the scientific background and rationale for the investigation being reported	2
Objectives	3	State specific objectives, including any prespecified hypotheses	3
**Methods**			
Study design	4	Present key elements of study design early in the paper	3
Setting	5	Describe the setting, locations, and relevant dates, including periods of recruitment, exposure, follow-up, and data collection	3
Participants	6	(*a*) Give the eligibility criteria, and the sources and methods of selection of participants. Describe methods of follow-up	3
		(*b*) For matched studies, give matching criteria and number of exposed and unexposed	NA
Variables	7	Clearly define all outcomes, exposures, predictors, potential confounders, and effect modifiers. Give diagnostic criteria, if applicable	4
Data sources/ measurement	8*	For each variable of interest, give sources of data and details of methods of assessment (measurement). Describe comparability of assessment methods if there is more than one group	3, 4
Bias	9	Describe any efforts to address potential sources of bias	4
Study size	10	Explain how the study size was arrived at	NA
Quantitative variables	11	Explain how quantitative variables were handled in the analyses. If applicable, describe which groupings were chosen and why	7
Statistical methods	12	(*a*) Describe all statistical methods, including those used to control for confounding	7
		(*b*) Describe any methods used to examine subgroups and interactions	8
		(*c*) Explain how missing data were addressed	7
		(*d*) If applicable, explain how loss to follow-up was addressed	-
		(*e*) Describe any sensitivity analyses	9
**Results**			
Participants	13*	(a) Report numbers of individuals at each stage of study—eg numbers potentially eligible, examined for eligibility, confirmed eligible, included in the study, completing follow-up, and	9

		analysed	
		(b) Give reasons for non-participation at each stage	-
		(c) Consider use of a flow diagram	9
Descriptive data	14*	(a) Give characteristics of study participants (eg demographic, clinical, social) and information on exposures and potential confounders	9
		(b) Indicate number of participants with missing data for each variable of interest	-
		(c) Summarise follow-up time (eg, average and total amount)	9
Outcome data	15*	Report numbers of outcome events or summary measures over time	10

Main results	16	(*a*) Give unadjusted estimates and, if applicable, confounder-adjusted estimates and their precision (eg, 95% confidence interval). Make clear which confounders were adjusted for and why they were included	10
		(*b*) Report category boundaries when continuous variables were categorized	NA
		(*c*) If relevant, consider translating estimates of relative risk into absolute risk for a meaningful time period	-
Other analyses	17	Report other analyses done—eg analyses of subgroups and interactions, and sensitivity analyses	11
**Discussion**			
Key results	18	Summarise key results with reference to study objectives	12
Limitations	19	Discuss limitations of the study, taking into account sources of potential bias or imprecision. Discuss both direction and magnitude of any potential bias	15
Interpretation	20	Give a cautious overall interpretation of results considering objectives, limitations, multiplicity of analyses, results from similar studies, and other relevant evidence	14
Generalisability	21	Discuss the generalisability (external validity) of the study results	15
**Other information**			
Funding	22	Give the source of funding and the role of the funders for the present study and, if applicable, for the original study on which the present article is based	Title page

Supplementary Figure S1. Probability of mortality

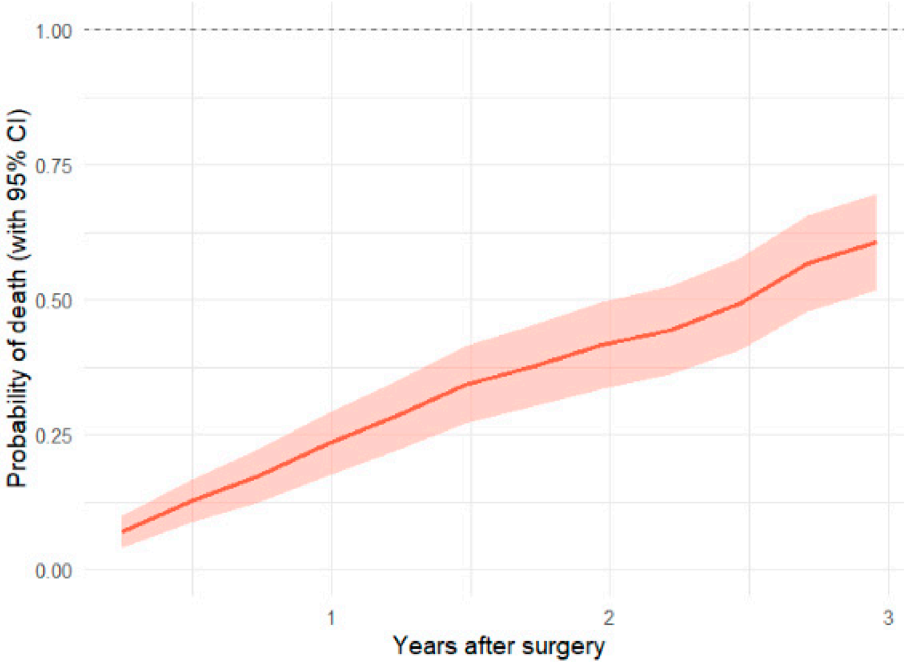

Supplementary Table S2. Probability of mortality

	Total, n	Death, n	Death probability (95% CI)
Postoperative 3 months	302	21	0.06 (0.04, 0.09)
Postoperative 6 months	271	34	0.12 (0.08, 0.16)

Postoperative 9 months	226	39	0.17 (0.12, 0.22)
Postoperative 12 months	207	48	0.23 (0.17, 0.28)
Postoperative 15 months	186	53	0.28 (0.22, 0.34)
Postoperative 18 months	167	57	0.34 (0.26, 0.41)
Postoperative 21 months	159	60	0.37 (0.3, 0.45)
Postoperative 24 months	147	61	0.41 (0.33, 0.49)
Postoperative 27 months	140	62	0.44 (0.36, 0.52)
Postoperative 30 months	130	64	0.49 (0.4, 0.57)
Postoperative 33 months	120	68	0.56 (0.47, 0.65)
Postoperative 36 months	117	71	0.60 (0.51, 0.69)

Supplementary Figure S2. Probability of new nursing home admission

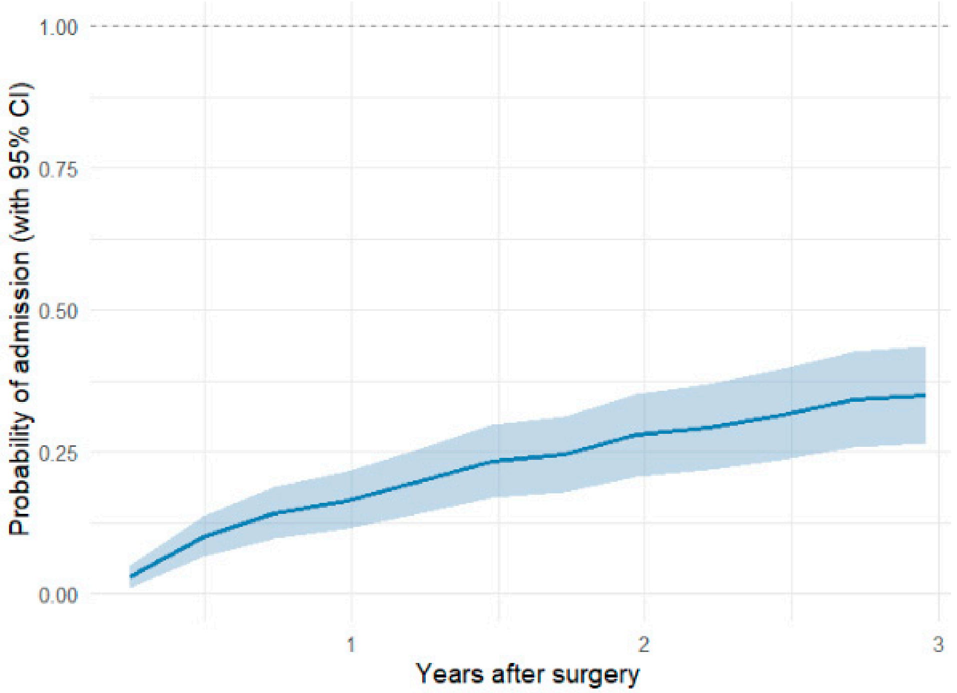

Supplementary Table S3. Probability of new nursing home admission

	Total, n	New nursing home admission, n	New nursing home admission probability (95% CI)
Postoperative 3 months	302	9	0.02 (0.01, 0.04)
Postoperative 6 months	271	27	0.09 (0.06, 0.13)
Postoperative 9 months	226	32	0.14 (0.09, 0.18)
Postoperative 12 months	207	34	0.16 (0.11, 0.21)
Postoperative 15 months	186	37	0.19 (0.14, 0.25)
Postoperative 18 months	167	39	0.23 (0.16, 0.29)
Postoperative 21 months	159	39	0.24 (0.17, 0.31)

Postoperative 24 months	147	41	0.27 (0.20, 0.35)
Postoperative 27 months	140	41	0.29 (0.21, 0.36)
Postoperative 30 months	130	41	0.31 (0.23, 0.39)
Postoperative 33 months	120	41	0.34 (0.25, 0.42)
Postoperative 36 months	117	41	0.35 (0.26, 0.43)

Supplementary Figure S3. Standard mortality ratio

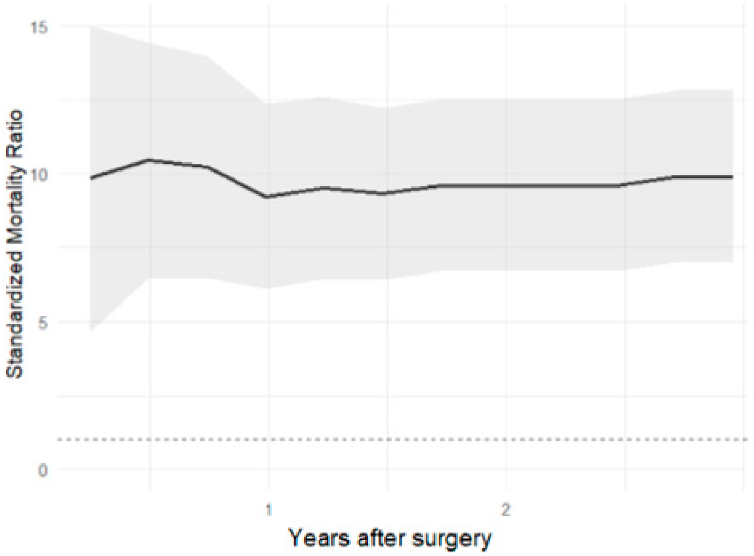

Solid lines indicate the ratio, and gray shaded areas indicate 95% confidence intervals.

Supplementary Table S4. Standard mortality ratio

	Observed death, n	Expected death, n	Standard mortality ratio (95% CI)
Postoperative 3 months	14	1.42	9.85 (4.69, 15.0)
Postoperative 6 months	26	2.49	10.4 (6.42, 14.4)
Postoperative 9 months	28	2.74	10.2 (6.43, 13.9)
Postoperative 12 months	33	3.57	9.21 (6.07, 12.3)
Postoperative 15 months	36	3.79	9.49 (6.39, 12.5)
Postoperative 18 months	39	4.19	9.30 (6.38, 12.2)
Postoperative 21 months	41	4.27	9.59 (6.65, 12.5)
Postoperative 24 months	41	4.27	9.59 (6.65, 12.5)
Postoperative 27 months	41	4.27	9.59 (6.65, 12.5)
Postoperative 30 months	41	4.27	9.59 (6.65, 12.5)
Postoperative 33 months	44	4.45	9.88 (6.96, 12.8)
Postoperative 36 months	44	4.45	9.88 (6.96, 12.8)

Supplementary Table S5. Univariate analysis of risk factors associated with mortality.

		Total (n = 355)	Survivors (n = 278)	Death (n = 77)	p-value
Pre-fracture ambulatory ability, n (%)	No aid	173 (48.7)	138 (49.6)	35 (45.5)	0.03
	Cane	63 (17.7)	55 (19.8)	8 (10.4)	
	Walker	70 (19.7)	53 (19.1)	17 (22.1)	
	Wheelchair.	49 (13.8)	32 (11.5)	17 (22.1)	
Pre-fracture ambulatory ability, n	Dependent	49 (13.8)	32 (11.5)	17 (22.1)	0.02
Pre-fracture resistance, n (%)	Own home (living alone)	59 (16.6)	45 (26.0)	14 (33.3)	0.33
	Own home (non-living alone)	156 (43.9)	128 (74.0)	28 (66.7)	
	Nursing home	125 (35.2)	95 (34.2)	30 (39.0)	
	Hospital	15 (4.2)	10 (3.6)	5 (6.5)	
Pre-fracture life assistance, n (%)	No assistance	59 (16.6)	45 (16.2)	14 (18.2)	0.73
Public assistance recipient, n (%)		30 (8.5)	27 (9.7)	3 (3.9)	
History of hip fracture, n (%)		29 (8.2)	22 (7.9)	7 (9.1)	0.22
Albumin, g/dL		3.7 (3.3, 3.9)	3.7 (3.4, 4.0)	3.4 (3.1, 3.8)	3.02
Geriatric Nutritional Risk Index, n (%)	Major risk (GNRI <82)	74 (20.8)	44 (15.8)	30 (39.0)	< 0.01
	Moderate risk (GNRI 82 to <92)	100 (28.2)	81 (29.1)	19 (24.7)	
	Low risk (GNRI 92 to ≤98)	70 (19.7)	59 (21.2)	11 (14.3)	
	No risk (GNRI >98)	111 (31.3)	94 (33.8)	17 (22.1)	

Data are presented as number (%) or median (interquartile range). Geriatric Nutritional Risk Index, GNRI

Supplementary Table S6. Univariate analysis of risk factors associated with new nursing home admission

		Total (n = 230)	Not new admitted (n=189)	New admitted (n=41)	p-value
BMI, kg/m^2		19.9 (17.5, 23.4)	20.0 (17.6, 23.6)	19.4 (17.4, 23.2)	0.67
Pre-fracture ambulatory ability, n (%)	No aid	133 (57.8)	113 (59.8)	20 (48.8)	0.47
	Cane	51 (22.2)	40 (21.2)	11 (26.8)	
	Walker	32 (13.9)	24 (12.7)	8 (19.5)	
	Wheelchair.	14 (6.1)	12 (6.3)	2 (4.9)	
Pre-fracture ambulatory ability, n	Dependent	216 (93.9)	12 (6.3)	2 (4.9)	1.00
	No aid	133 (57.8)	113 (59.8)	20 (48.8)	0.47
Pre-fracture resistance, n (%)	Own home (living alone)	14 (6.1)	46 (24.3)	13 (31.7)	0.74

	Own home (non-living alone)	59 (25.6)	131 (69.3)	25 (61.0)	
	Hospital	156 (67.8)	12 (6.3)	3 (7.3)	
Pre-fracture life assistance, n (%)	No assistance	15 (6.5)	46 (24.3)	13 (31.7)	0.33
Public assistance recipient, n (%)		59 (25.7)	14 (7.4)	4 (9.8)	0.62
History of hip fracture, n (%)		18 (7.8)	8 (4.2)	2 (4.9)	0.69
Bilateral hip fractures, n (%)		11 (4.8%)	4 (2.1)	7 (17.1)	0.11
Fracture classification	Trochanteric fracture	10 (4.3)	88 (46.6)	25 (61.0)	0.12
Charlson comorbidity index		55 (23.9)	1.0 (0.0, 3.0)	1.0 (1.0, 2.0)	0.18
Albumin, g/dL		1 (0, 2)	3.7 (3.3, 4.0)	3.7 (3.4, 3.9)	0.71
Geriatric Nutritional Risk Index, n (%)	Major risk (GNRI <82)	3.7 (3.3, 4.0)	40 (21.2)	7 (17.1)	0.23
	Moderate risk (GNRI 82 to <92)	47 (20.4)	47 (24.9)	16 (39.0)	
	Low risk (GNRI 92 to ≤98)	63 (27.4)	35 (18.5)	4 (9.8)	
	No risk (GNRI >98)	39 (17.0)	67 (35.4)	14 (34.1)	
Surgery from injury, days		81 (35.2)	5.0 (2.0, 11.0)	5.0 (2.0 ,11.0)	0.74

Data are presented as number (%) or median (interquartile range). Bilateral hip fractures indicate patients who sustained hip fractures on both sides (including those with a prior hip fracture and a subsequent contralateral fracture). Geriatric Nutritional Risk Index, GNRI.

Supplementary Table S7. Logistic regression models for mortality

		Odds ratio (95% CI)	p-value
Model 2	Age	0.96 (0.93, 1.00)	0.08
	Sex, man	0.48 (0.26–0.92)	0.02
Model 3	Age	0.96 (0.92–1.00)	0.06
	Sex, man	0.49 (0.25–0.95)	<0.01
	Early surgery	2.10 (1.10–40.27)	0.03
	CCI	0.78 (0.66–0.91)	<0.01
	GNRI: no/low risk	1.80 (1.04–3.11)	0.04

Charlson comorbidity index, CCI; Geriatric Nutritional Risk Index, GNRI

Supplementary Table S8. Logistic regression models for new nursing home admission

		Odds ratio (95% CI)	p-value
Model 2	Age	0.92 (0.87, 0.97)	<0.01
	Sex, man	1.58 (0.61, 4.94)	0.38
Model 3	Age	0.94 (0.89, 1.00)	0.07
	Sex, man	1.46 (0.51, 4.84)	0.49
	Dementia	0.53 (0.23, 1.25)	0.14
	Living with someone before fracture	1.52 (0.64, 3.51)	0.33
	Ambulatory ability regain at postoperative 3 months	2.88 (1.29, 6.51)	0.01

Supplementary Table S9. Subgroup analyses of the Cox regression model for mortality

		Covariate	Hazard ratio (95%CI)	p-value
Sex	male	Age	0.94 (0.90, 0.99)	0.04
		CCI	1.17 (0.88, 1.56)	0.27
		GNRI no/low risk	1.50 (0.53, 4.25)	0.44
		Early surgery	0.74 (0.28, 1.89)	0.53
Prefracture independent ambulatory ability	Prefracture mobility independent	Sex	1.09 (0.23, 4.96)	0.91
		Age	1.02 (0.95, 1.08)	0.54
		CCI	0.90 (0.65, 1.24)	0.54
		GNRI no/low risk	0.97 (0.34, 2.74)	0.97
		Early surgery	1.31 (0.54, 3.12)	0.54
Dementia	Demented	Sex	1.87 (0.74, 4.75)	0.18
		Age	0.94 (0.90, 0.99)	0.02
		CCI	1.17 (0.94, 1.47)	0.14
		GNRI no/low risk	0.74 (0.39, 1.41)	0.37
		Early surgery	0.73 (0.37, 1.44)	0.37
GNRI	GNRI no/low risk	Sex	1.50 (0.53, 4.25)	0.44
		Age	0.96 (0.92, 1.01)	0.16
		CCI	0.89 (0.70, 1.12)	0.32
		Early surgery	2.27 (1.12, 4.59)	0.02
Side	Bilateral fracture	Sex	0.75 (0.18, 3.03)	0.68
		Age	1.08 (1.01, 1.14)	0.01
		CCI	1.20 (0.91, 1.60)	0.18

		GNRI no/low risk	4.85 (0.98, 3.90)	0.05
		Early surgery	0.28 (0.07, 1.06)	0.06
Living with someone	Living alone	Sex	0.86 (0.27, 2.75)	0.80
		Age	1.06 (0.99, 1.13)	0.05
		CCI	1.19 (0.87, 1.63)	0.26
		GNRI no/low risk	1.07 (0.40, 2.88)	0.87
		Early surgery	1.04 (0.37, 2.94)	0.93
Diagnosis	Trochanteric fracture	Sex	1.34 (0.59, 3.05)	0.47
		Age	1.00 (0.97, 1.04)	0.65
		CCI	0.99 (0.80, 1.22)	0.96
		GNRI no/low risk	0.95 (0.50, 1.81)	0.89
		Early surgery	0.44 (0.22, 0.87)	0.01

Charlson comorbidity index, CCI; Geriatric Nutritional Risk Index, GNRI

Supplementary Table S10. Subgroup analyses of the Cox regression model for new nursing home admission

		Covariate	Hazard ratio (95%CI)	p-value
Sex	Male	Age	0.98 (0.84, 1.12)	0.74
		Dementia	0.93 (0.09, 9.36)	0.95
		Living with someone	0.84 (0.12, 5.91)	0.86
		Ambulatory ability regain achieved	0.19 (0.01, 1.91)	0.16
Prefracture independent ambulatory ability	Independent	Age	0.81 (0.58, 1.12)	0.21
		Sex	458, (0.00, Inf)	1.00
		Dementia	22,4 (0.00, Inf)	1.00
		Living with someone	0.00 (0.00, Inf)	1.00
		Ambulatory ability regain achieved	0.20 (0.01, 3.63)	0.28
Dementia	Demented	Age	1.04 (0.92, 1.15)	0.55
		Sex	0.93 (0.09, 9.36)	0.95
		Living with someone	0.70 (0.17, 2.77)	0.61
		Ambulatory ability regain achieved	0.89 (0.21, 3.60)	0.87
GNRI	GNRI no/low risk	Age	1.24 (0.93, 1.63)	0.14

		Sex	0.00 (0.00, 0–In)	1.00
		Dementia	2.96 (0.32, 7.33)	0.34
		Living with someone	2.96 (0.24, 5.63)	0.39
		Ambulatory ability regain achieved	0.43 (0.03, 4.96)	0.50
Side	Bilateral fractures	Age	0.99 (0.88, 1.11)	0.89
		Sex	0.00 (0.00, 0–In)	1.00
		Dementia	0.81 (0.09, 6.62)	0.84
		Living with someone	21,1 (0.00, Inf)	1.00
		Ambulatory ability regain achieved	0.45 (0.04, 5.01)	0.52
Living with someone	Living alone	Age	0.92 (0.82, 1.01)	0.08
		Sex	1.00 (0.14, 7.07)	1.00
		Dementia	1.37 (0.33, 5.59)	0.67
		Ambulatory ability regain achieved	2.15 (0.53, 8.71)	0.28
Diagnosis	Trochanteric fracture	Age	0.99 (0.89, 1.09)	0.90
		Sex	1.02 (0.14, 6.94)	0.99
		Dementia	0.87 (0.22, 3.37)	0.84
		Living with someone	2.65 (0.66, 0.62)	0.17
		Ambulatory ability regain achieved	1.10 (0.28, 4.24)	0.89

Charlson comorbidity index, CCI; Geriatric Nutritional Risk Index, GNRI Inf, Infinity

Supplementary Table S11. Sensitivity analyses of the Cox regression model for mortality

	Covariate	Hazard ratio (95% CI)	p-value
Exclude death ≤30 days	Sex	0.83 (0.55, 1.24)	0.37
	Age	1.00 (0.98, 1.03)	0.41
	CCI	1.00 (0.90, 1.12)	0.87
	GNRI: major risk	1.36 (0.85, 2.18)	0.19
	Early surgery	2.44 (1.70, 3.50)	<0.01
Exclude patients with dementia	Sex	0.79 (0.51, 1.22)	0.30
	Age	1.00 (0.98, 1.03)	0.57
	CCI	0.97 (0.85, 1.10)	0.66

	GNRI: major risk	1.22 (0.73, 2.06)	0.44
	Early surgery	2.48 (1.66, 3.71)	<0.01
Exclude patients who used a wheelchair before the fracture	Sex	0.87 (0.58, 1.30)	0.52
	Age	1.01 (0.99, 1.03)	0.34
	CCI	1 (0.89, 1.11)	1.00
	GNRI: major risk	1.42 (0.90, 2.23)	0.13
	Early surgery	2.32 (1.62, 3.31)	<0.01

Charlson comorbidity index, CCI; Geriatric Nutritional Risk Index, GNRI

Supplementary Table S12. Sensitivity analyses of the Cox regression model for new nursing home admission

	Covariate	Hazard ratio (95% CI)	p-value
Exclude new nursing home admission ≤30 days	Age	1.06 (1.00, 1.11)	0.02
	Sex	0.81 (0.30, 2.16)	0.67
	Dementia	1.50 (0.76, 2.97)	0.23
	Living with someone before fracture	0.80 (0.40, 1.61)	0.55
	Ambulatory ability regain at postoperative 3 months	0.30 (0.15, 0.60)	<0.01
Exclude patients with public assistance recipient	Age	1.08 (1.02, 1.14)	<0.01
	Sex	0.74 (0.25, 2.18)	0.59
	Dementia	1.56 (0.79, 3.08)	0.19
	Living with someone before fracture	0.95 (0.45, 1.98)	0.89
	Ambulatory ability regain at postoperative 3 months	0.35 (0.17, 0.70)	<0.01
Dataset with multiple imputation for missing data	Age	1.06 (1.01, 1.12)	0.01
	Sex	0.87 (0.32, 2.31)	0.78
	Dementia	1.81 (0.91, 3.59)	0.08
	Living with someone before fracture	0.87 (0.42, 1.77)	0.70
	Ambulatory ability regain at postoperative 3 months	0.38 (0.19, 0.78)	<0.01
